# RHDV2 epidemic in UK pet rabbits. Part 2: PCR results and correlation with vaccination status

**DOI:** 10.1111/jsap.13180

**Published:** 2020-07-26

**Authors:** F. M. Harcourt‐Brown, N. Harcourt‐Brown, L. M. Joudou

**Affiliations:** ^1^ North Yorkshire UK; ^2^ Filavie Laboratory Roussay France

## Abstract

**Objective:**

To report PCR results and vaccination status of rabbits with rabbit haemorrhagic disease following an investigation into sudden or unexpected death.

**Materials and Methods:**

PCR testing for RHDV2 and RHDV1 was performed on rabbit liver samples at two laboratories. Laboratory A reported results as positive or negative; Laboratory B reported results quantitatively as RNA copies *per* mg liver, categorised as negative, inconclusive or positive. The vaccination status of rabbits with both histopathological features of rabbit haemorrhagic disease and positive PCR test results were collated.

**Results:**

PCR results matched histopathological findings in 188 of 195 (96%) cases. Seven individuals showed equivocal results, all of which had histopathological features of RHD but three tested PCR‐negative and four results conflicted between laboratories. RHDV2 was the serotype detected in all PCR‐positive cases. Histological features of rabbit haemorrhagic disease and PCR test results were positive in 125 rabbits; 51 unvaccinated, 56 in‐date with Nobivac Myxo‐RHD and 13 vaccinated against RHDV2 – although nine of these were vaccinated within 10 days of death.

**Clinical Significance:**

PCR testing complements histopathology in cases of sudden death in rabbits by confirming the diagnosis and identifying virus serotype, but there can be false negatives. Although RHDV2 is currently prevalent in UK pet rabbits, vaccination against both RHDV1 and RHDV2 is recommended. Failures of RHDV2 vaccine are infrequent.

## INTRODUCTION

Rabbit haemorrhagic disease (RHD) is caused by a non‐enveloped single‐stranded positive‐sense RNA virus (RHDV) that belongs to the genus *Lagovirus* (Abrantes *et al*. 2012, Le Gall‐Reculé *et al*. 2017). In 2010, a new serotype of RHDV (RHDV2) that is phylogenetically, antigenically and serotypically distinct from previously identified strains of RHDV (RHDV1) was identified in France (Le Gall‐Reculé *et al*. 2011, 2013). After its discovery, RHDV2 spread rapidly through Europe. In the UK, RHDV2 was first reported in 2014 (Westcott & Choudhury 2015). By 2016, outbreaks were killing rabbits in rescue centres, breeding colonies and show rabbits as well as individual pets (Harcourt‐Brown 2016, Rocchi & Dagleish 2018). Immunity against RHDV1 does not protect against RHDV2 (Le Gall‐Reculé *et al*. 2013, Peacock *et al*. 2017) and deaths occurred in rabbits that were vaccinated against RHDV1 with Nobivac Myxo‐RHD (MSD Animal Health), which was the only vaccine available in the UK at the outset of the RHDV2 epidemic. In 2016, adjuvanted inactivated vaccines against RHDV2, such as Eravac (Hipra Laboratories, Spain) and Filavac VHD K C + V (Filavie Laboratories, France) started to be imported (Saunders 2016) and, by 2018, both vaccines had product licences and were available in the UK.

A diagnosis of RHD can be made from the clinical history, gross *postmortem* examination and histopathology (Part 1). PCR testing recognises viral RNA and identifies the RHDV serotype (RHDV1 or RHDV2). Here we present results of PCR testing and vaccination status of rabbits that died from RHD.

## MATERIALS AND METHODS

From November 2016 to December 2018, an investigation into sudden and unexpected deaths in pet rabbits was conducted and tissue samples were collected for histopathology during *postmortem* examination by veterinary practitioners (Harcourt‐Brown *et al*. 2020 [Part 1]). Additional liver samples were collected, frozen and stored at the veterinary practice before submission for PCR testing at one or both of two laboratories. Both laboratories were blinded to the histopathology results.

At the outset of the investigation, only frozen liver samples from rabbits that showed hepatocellular necrosis consistent with RHD were submitted for PCR testing. These samples were sent directly from the veterinary practice to a commercial laboratory (Laboratory A). From April 2017, PCR testing was conducted at a pharmaceutical laboratory (Laboratory B) and frozen liver samples were requested for PCR testing from all the rabbits that died unexpectedly, instead of just those with histopathological signs of RHD. Samples destined for Laboratory B were sent to us, where they were stored in a domestic freezer and dispatched in batches of 35‐55 samples to Laboratory B. From August 2017 until the end of the investigation, veterinarians were requested to submit two pieces of frozen liver instead of a single piece, so a reserve sample was available for cases with equivocal PCR results.

At Laboratory A, RNA was prepared using a commercial kit (MagNA Pure 96 DNA and viral NA small volume kit, Roche) according to manufacturer instructions. Real‐time PCR for the detection of RHDV/RHDV1 (Gall & Schirrmeier 2006) and RHDV2 (Duarte *et al*. 2015) were carried out as described. Results were reported as positive or negative for RHDV1 and RHDV2. At Laboratory B, total RNA was extracted using Nucleospin RNA virus kit (Macherey‐Nagel, Düren, Germany) according to manufacturer instructions. Real‐time reverse transcriptase quantitative PCR for the detection of RHDV2 described by Le Minor *et al*. (2019) were carried out. The primers and probe sequences for the RHDV2 RT‐qPCR were designed by Scanelis (Toulouse, France) based on conserved regions evidenced on the alignment of VP60 complete RHDV2 sequences. Results for Laboratory B were reported quantitatively as the amount of RNA copies *per* mg liver. The samples were only tested for RHDV2, except for two cases with hepatocellular necrosis consistent with RHD but low viral loads of RHDV2. These samples were subsequently tested for RHDV1 at Laboratory B.

The quantitative PCR test results for RHDV2 were divided into three categories depending on the likelihood of RHDV2 as the cause of death of the rabbit. The categories were:

**“Positive”**: Samples with PCR test results above 10^7^ RNA copies/mg liver were positive for viral RHDV2 RNA and the viral load was high enough to cause death from RHD. The cut‐off point of 10^7^ RNA copies/mg liver was based on research conducted at Laboratory B and is in accordance with results of investigations into RHDV2 infection (Dalton *et al*. 2018, Neimanis *et al*. 2018, Le Moullec *et al*. 2019).

**“Inconclusive”:** Samples with PCR test results between the limit of quantification (1.9 × 10^3^) and 10^7^ RNA copies/mg were positive with medium‐low load of RHDV2 RNA, but not high enough to cause certain death from RHD. Additional information from clinical finding, gross *postmortem* examination and histopathology was necessary to make a diagnosis.

**“Negative”:** Samples with test results below the quantification limit of 1.9 × 10^3^ RNA copies/mg liver were negative or inconclusive for RHDV2 RNA. The quantification limit is the lowest amount that can be reliably quantified with an acceptable level of precision and trueness (Hougs *et al*. 2017) so viral RNA was either absent or present in tiny quantities these samples.

At the end of the investigation, 19 reserve liver samples with negative or inconclusive results from Laboratory B were sent to Laboratory A for PCR testing because it was not known whether they would be reported as positive or negative by another laboratory. A twentieth reserve liver sample was submitted from a case that tested negative for RHDV2 at Laboratory B but showed hepatocellular necrosis consistent with RHD on histopathology.

After the investigation was concluded and all PCR results were available, seven cases with conflicting results between histopathological findings and PCR results from one or both laboratories were reviewed. New tissue sections were cut, stained and examined by two pathologists who were blinded to the original histopathology report, PCR results and each other's assessment.

## RESULTS

### PCR results

PCR testing was performed on the liver of 195 of 300 rabbits. The results and the laboratory that carried out tests are shown in Table 1. The cause of death of these rabbits is shown in Table 2; the diagnosis was made from the history, clinical findings, gross *postmortem* lesions and histopathology. RHDV2 was identified in all 128 PCR positive results and in 22 cases with inconclusive results from Laboratory B. The 37 cases that were PCR‐tested for RHDV1 were all negative.

**Table 1:** Summary of PCR results from Laboratory A and Laboratory B

Where PCR test was conducted	Number of samples	Result		
		Positive	Negative	Inconclusive (from Laboratory B only)
Laboratory A	15			
RHDV1		0	15	
RHDV2		13	2	
Laboratory B	160			
RHDV1		0	2	
RHDV2		112	42	6
Both laboratory A and B	20			
RHDV1		0	20	
RHDV2				
Laboratory A		3	17	
Laboratory B			4	16
TOTAL	195			
RHDV1		0	37	
RHDV2		125	48	22
Conflicting results between Laboratory A and B		3		

RHD **r**abbit haemorrhagic disease

**Table 2:** Cause of death in 195 rabbits that were PCR‐tested for RHDV2

	Cause of death	
PCR negative	Undetermined	15
	Non RHD liver disease (liver lobe torsion, hepatic coccidiosis)	3
	Lower respiratory tract disease (histiocytosis, lung abscess, pulmonary embolism, acute intra‐alveolar haemorrhage)	4
	Enteric disease (enterotoxaemia, coccidiosis, intussusception, mucoid enteropathy)	4
	Heart and circulatory disease	7
	Sepsis/septicaemia	2
	Renal disease	4
	Pancreatitis	1
	Choking or aspiration pneumonia	2
	Necrotic tongue	1
	Myxomatosis	1
	RHD from histopathology (Cases 5–7, Table 3)	3
PCR inconclusive	Undetermined	6
	Non RHD liver disease (liver lobe torsion, hepatic coccidiosis)	2
	Pneumonia	2
	Enterotoxaemia	1
	Heart and circulatory disease	2
	Sepsis/septicaemia	2
	Neoplasia (uterine adenocarcinoma)	1
	Trauma	1
	Pancreatitis	1
	Choking or aspiration pneumonia	1
	RHD from histopathology (Case 3, Table 3)	1
PCR positive	RHD from histopathology	125
Conflicting results between laboratories	RHD from histopathology (Case 4, Table 3)	1
	Possible RHD from histopathology (Cases 1 and 2, Table 3)	2
	TOTAL	195

RHD **r**abbit haemorrhagic disease

The combined PCR test results from both Laboratory A and B matched the histopathological findings in 188 of 195 (96%) cases. The remaining seven (4%) samples showed equivocal results because there was conflict between histopathology and PCR testing or between PCR results from Laboratory A and B (Table 3).

**Table 3:** Details of cases with conflicting results between histopathology and PCR or between Laboratory A and Laboratory B

Case	Histopathology report (abridged)	Results from Laboratory A	Result from Laboratory B	Vaccination status
1	Coccidial infection in the liver, which is much more severe in some areas than in others. Focal parenchymal inflammation and cell degeneration, not the typical distribution of lesions of RHD. Alveolar oedema. Scattering of karyorrhexis and neutrophils in spleen with some macrophage activity. No abnormality detected in heart kidney and pancreas **RHD POSSIBLE** *	**‐**ve RHDV1 **+ve RHDV2**	**Inconclusive for RHDV2** (3.53 × 10^6^ RNA copies/mg liver)	Unvaccinated
2	**ORIGINAL REPORT** Congestion in liver and normal overall lobular architecture with some vacuolation of hepatocytes. Pulmonary congestion and collapse. Alveolar oedema. No significant pathology in thymus, spleen, pancreas, kidney or heart. **NOT RHD.** **REVIEW OF NEW SECTIONS** Foci of necrosis and inflammation but overall liver mildly affected. Concurrent congestion and fibrin thrombi in sinusoids and central veins. Glomerular congestion with occasional thrombi in some sections of kidney **POSSIBLE PERACUTE RHD**	‐ve RHDV1 **+ve RHDV2**	**Inconclusive for RHDV2** (4,56 × 10^4^ RNA copies/mg liver)	Unvaccinated
3	Severe sub‐acute hepatic necrosis. Moderate congestion in lungs, kidney and spleen. Adrenal gland, mesenteric lymph node, myocardium and sections of caecum/colon are unremarkable. **CONSISTENT WITH SUBACUTE RHD** *		‐ve for RHDV1 **Inconclusive for RHDV2** (1.80 × 10^4^ RNA copies/mg liver)	Unvaccinated
4	Widespread hepatocellular necrosis. Mild interstitial lymphoplasmacytic inflammation in kidney unrelated to death of animal. No abnormality in heart, spleen, lungs and trachea **CONSISTENT WITH RHD** *	‐ve RHDV1 **+ve RHDV2**	**‐ve RHDV2**	Unvaccinated
5	Marked widespread acute hepatic necrosis. Glomerular thrombosis, Splenic congestion and haemorrhage. Moderate pulmonary congestion and mild alveolar oedema. Congested thymus. Congested sub‐mucosal vessels in trachea. Unremarkable myocardium **CONSISTENT WITH RHD** *		‐ve RHDV1 **‐ve RHDV2**	Unvaccinated
6	Severe periportal acute coagulative hepatocellular necrosis. Canalicular cholestasis. Bile duct proliferation with peribiliary fibrosis (? previous coccidiosis). Moderate pulmonary congestion and patchy, mild alveolar oedema Haemorrhagic red pulp In spleen with increased neutrophils. Small areas of myocardial fibrosis. No significant changes in kidney **CONSISTENT WITH RHD** *	**‐**ve RHDV1 **‐ve RHDV2**		Nobivac Myxo‐RHD 14 days earlier
7	Some autolysis of tissues. Congestion and marked acute hepatocellular necrosis. Congested spleen. Unremarkable kidney. Patchy alveolar oedema. Multifocal haemorrhage in thymus. **CONSISTENT WITH RHD** *		**‐ve RHDV2**	Filavac K C + V 8 months earlier. Myxo‐RHD 9 months earlier

RHD **r**abbit haemorrhagic disease

* Results from original histopathology report agree with conclusion of independent blinded review of new tissue sections by two experienced histopathologists

**FIG 1:**
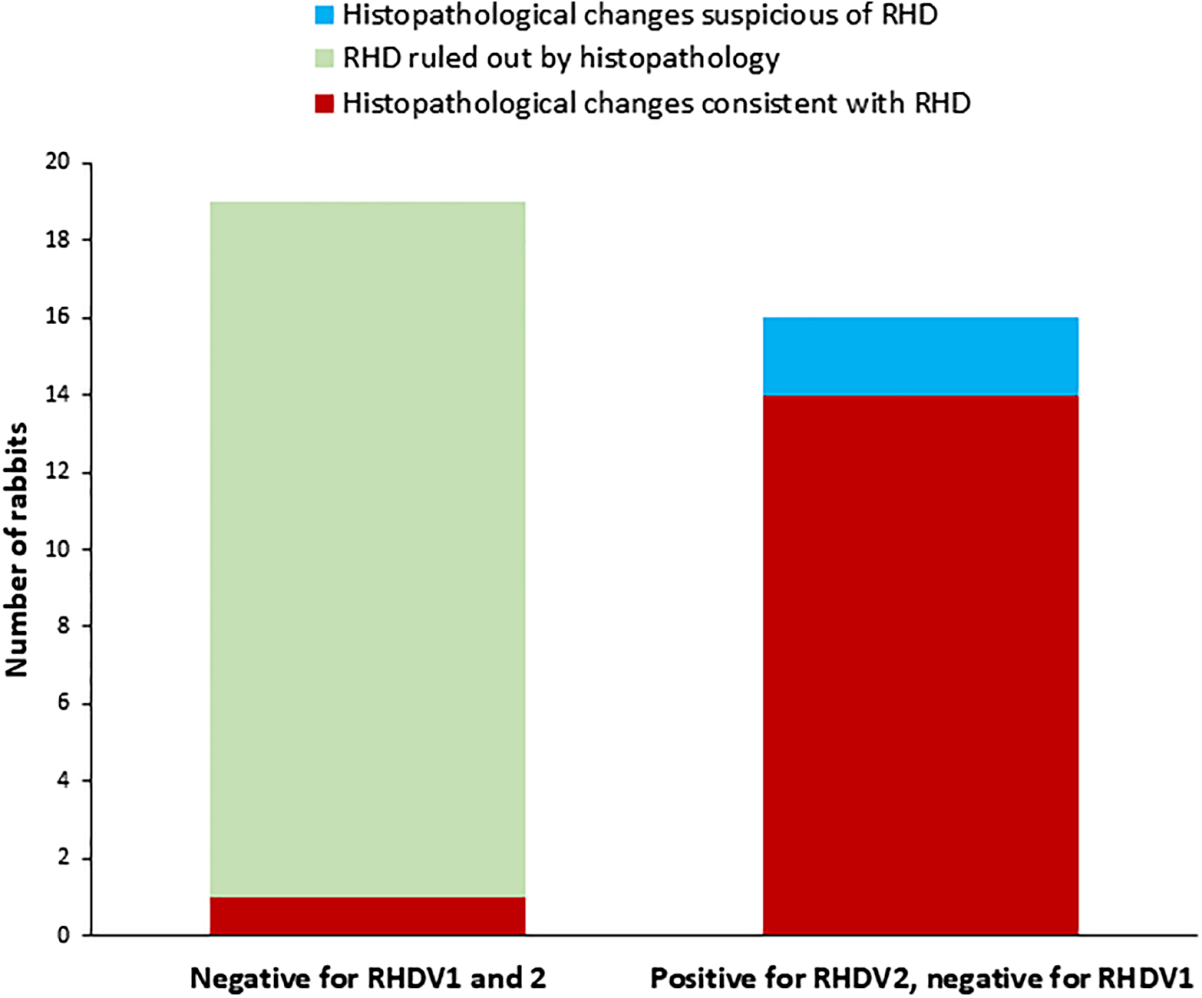
PCR results from Laboratory A in comparison with histopathology findings

At Laboratory A, RHDV2 positive results matched histopathological findings consistent with, or suspicious of, RHD in all 16 cases that were submitted (Fig. 1). Negative RHDV2 PCR results matched an alternative or undetermined cause of death in 18 of 19 cases. One case with histopathological features of RHD tested negative for RHDV1 and RHDV2 at Laboratory A (Case 6, Table 3).

**FIG 2:**
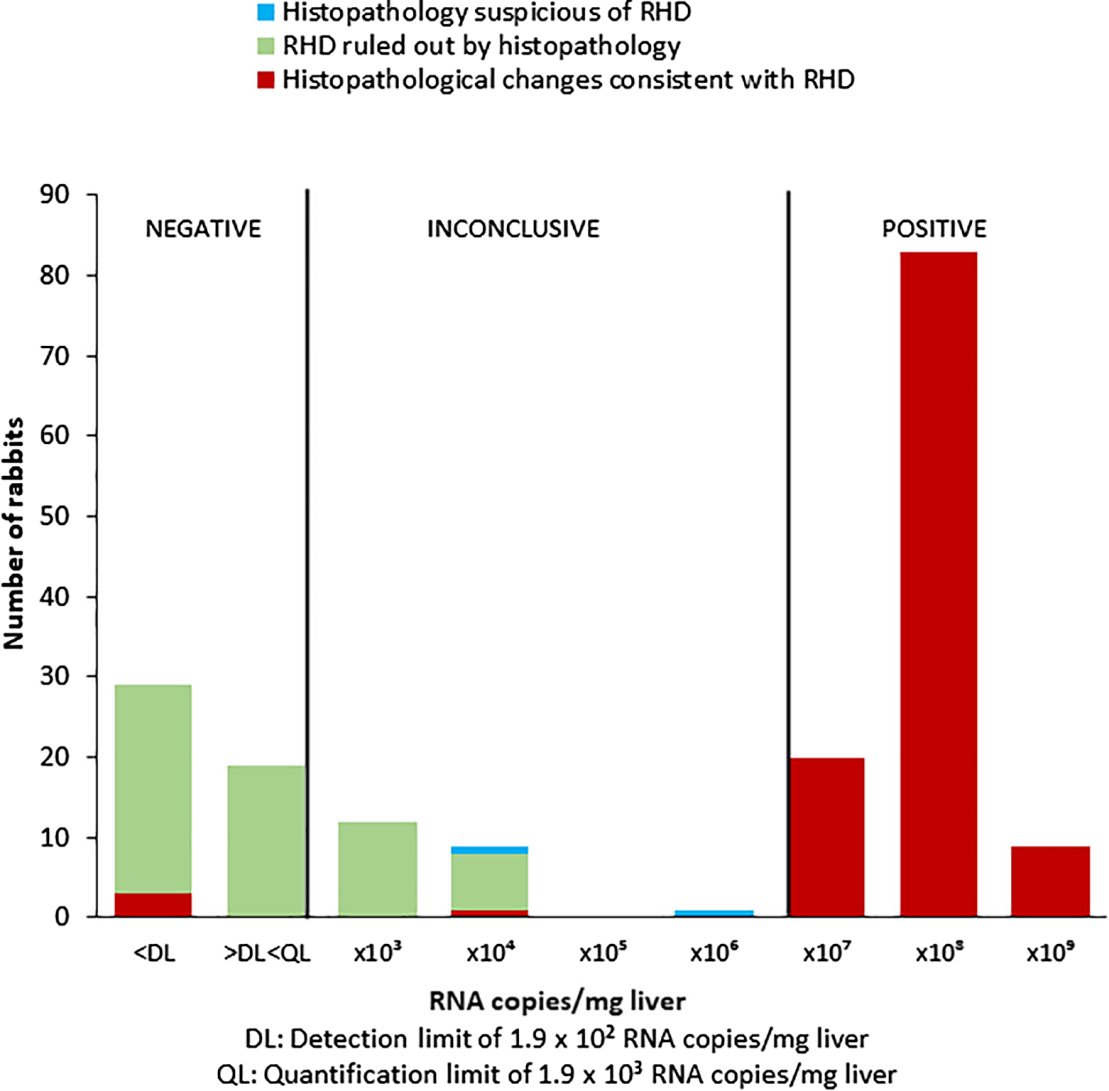
Categorisation of quantitative PCR test results for RHDV2 from Laboratory B in comparison with histopathology findings

At Laboratory B, there were histopathological changes consistent with RHD in all 112 cases categorised as PCR‐positive for RHDV2 (Fig. 2). There were histopathological findings consistent with, or suspicious of, RHD in three of 22 cases with inconclusive results (Cases 1‐3, Table 3) and in three cases that were categorised as negative (Cases 4.5 and 7, Table 3).

Twenty duplicate liver samples were sent to both Laboratory A and B. Of these, 14 of 16 samples with inconclusive PCR test results from Laboratory B tested negative for RHDV2 at Laboratory A and the remaining two tested positive (Cases 1 and 2, Table 3). The sample from a rabbit with histopathological findings consistent with RHD but a negative PCR test result from Laboratory B tested PCR‐positive for RHDV2 at Laboratory A (Case 4, Table 3).

In the seven cases with conflicting results, histopathological review agreed with the original report in six cases and disagreed in one (Case 2, Table 3). On the original sections of this case, there was no evidence of RHD, but examination of new sections showed some histopathological features suspicious of RHD. After the review, all seven cases with conflicting results showed histopathological signs that were consistent with, or suspicious of RHD despite a negative or inconclusive PCR test result.

### Vaccination status

The vaccination status of 125 rabbits with both histopathological diagnosis of RHD and positive RHDV2 PCR result is shown in Table 4. RHD was diagnosed as the cause of death in 56 rabbits that were in‐date with Nobivac Myxo‐RHD ‐ *i.e*. the vaccine was given between 7 days and a year of death. Six of these rabbits were also vaccinated against RHDV2.

**Table 4:** Vaccination status of 125 rabbits with histopathological diagnosis of RHD and positive PCR result for RHDV2

Vaccination status	
Unknown	7
Unvaccinated	51
Vaccinated with Nobivac Myxo‐RHD only	54 (4 lapsed)
Vaccinated with Nobivac Myxo‐RHD and against RHDV2 [Filavac K C + V (5), Unknown brand (1)]	6 (2 RHDV2 vaccine given within 7 days of death)
Vaccinated against RHDV2 only [Filavac K C + V (7)]	7 (all given within 7 days of death)
TOTAL	125

RHD **r**abbit haemorrhagic disease

RHD was diagnosed in a further seven rabbits that were vaccinated against RHDV2 alone. Vaccination against RHDV2 was often in response to the death of another rabbit in the household and nine of 13 deaths in rabbits vaccinated against RHDV2 occurred within 7 days of their demise. The vaccination certificate was missing in another case; only verbal assurance of vaccination against RHDV2 was given by the vendor at the time of purchase.

It has been suggested that residues of RHDV2 vaccine virus could interfere with interpretation of PCR results (Carvalho *et al*. 2017) so the vaccination status of the 22 rabbits with inconclusive results is shown in Table 5.

**Table 5:** Vaccination status of 22 rabbits with inconclusive PCR results

Vaccination status	Number
Unknown	3
Unvaccinated	7
Nobivac Myxo‐RHD	6 (1 lapsed)
Nobivac Myxo‐RHD and RHDV2 vaccine	3
RHDV2 vaccine	3 (all within 10 days of death)
TOTAL	22

RHD **r**abbit haemorrhagic disease

## DISCUSSION

In this investigation, all RHDV2 PCR‐positive results matched histopathological findings that were consistent with, or suspicious for, RHD (Figs. 1 and 2), indicating that a positive PCR result is reliable. The cut‐off point of 10^7^ RNA copies/mg liver for a positive quantitative result was supported by the results.

The inconclusive results from Laboratory B were difficult to interpret and histopathology yielded additional information. This in agreement with Carvalho *et al*. (2017) who indicated that a diagnosis based on the detection of low levels of RHDV or RHDV2 RNA should be complemented by histopathology to elucidate infection status and other differential diagnoses should be considered. Three of the 22 rabbits with inconclusive quantitative PCR (Cases 1‐3, Table 3) showed histopathological features of RHD and it is probable that they died from the disease. In the remaining 19 rabbits with inconclusive results there were no histopathological signs of RHD, and another cause of death was found in 13 cases (Table 2). There are several possible explanations for medium‐low RHDV2 RNA loads in the liver of rabbits with no histopathological features of RHD. These rabbits could have been in the early stages of infection when viral replication in the liver has just begun and histopathological changes were minimal. Alternatively, non‐viable viruses may have been detected because PCR testing has a such a high sensitivity (Duarte *et al*. 2015). Medium‐low quantities of viral RNA could be residues from infection in rabbits that had recovered from the disease or due to exposure to RHDV2 in immunised animals. RHDV2 nucleic acid in these rabbits could signify a carrier status, although this has not been demonstrated in laboratory investigations. One study showed that RHDV RNA can persist in adult rabbits that overcome experimental infection for at least 15 weeks, although neither antigen nor infectious virus could be detected by antigen‐enzyme‐linked immunosorbent assay (ELISA) immunohistochemistry or experimental transmission studies (Gall *et al*. 2007).

Low amounts of vaccine virus are another explanation for medium‐low viral RNA, and three of 22 rabbits with inconclusive quantitative PCR results had been vaccinated against RHDV2 within 10 days of death. There are several hypotheses to explain the detection of vaccine virus in tissues. These include unintentional intravascular administration, increased permeability of the blood vessels near the inoculation site, the systemic distribution of viral RNA *via* phagocytes or the association of inactivated viral particles with erythrocytes (Eschbaumer *et al*. 2010, Steinrigl *et al*. 2010, De Leeuw *et al*. 2015).

Negative RHDV2 PCR results from Laboratory A and Laboratory B agreed with the histopathological findings in 44 of 48 cases but disagreed in four in which there were histopathological features of RHD. Overall, there were seven cases with histopathological features of RHD but with a negative or inconclusive PCR test result from one of the laboratories (Table 3). Histopathological features of RHD in these rabbits (Cases 3‐7, Table 3) suggest the cause of death was RHD and the negative PCR result was false. In three cases, the PCR result differed between laboratories (Cases 1, 2 and 4, Table 3). Differences in methodology or interpretation of the results could account for these conflicting results. One rabbit (Case 7, Table 3) was not tested for RHDV1 so it is possible that RHDV1 was its cause of death.

In this study, all 147 of 195 rabbits with a positive or inconclusive PCR test result showed viral RNA of RHDV2 serotype. All 37 rabbits that were tested for RHDV1 showed a negative result, suggesting that RHDV2 is replacing RHDV1 as the cause of RHD in the UK. This is the conclusion of several other authors (Westcott & Choudhury 2015, McGowan & Choudhury 2016, Marschang *et al*. 2018). Replacement of RHDV1 by RHDV2 is occurring throughout Europe, although RHDV1 was still detected in a small number of cases in Germany and the Netherlands in 2016/17 (Marschang *et al*. 2018). Replacement of RHDV1 by RHDV2 has major implications for vaccination protocols because vaccines against RHDV1 are not protective against RHVDV2 (Dalton *et al*. 2015, Reemers *et al*. 2017). In this study, 56 of 70 (80%) of the vaccinated rabbits that died from RHD were in‐date with Nobivac Myxo/RHD (Table 4) confirming that this vaccine is not protective against RHDV2. Additional vaccination specifically against RHDV2 is required. However, vaccination against RHDV1 is still necessary because the virus may could still be in the environment and in the wild rabbit population and act as a reservoir of infection to rabbits kept as pets.

In this study, 13 rabbits that were vaccinated against RHDV2 died from the disease (Table 4) but there were explanations for the vaccine failure in many of these cases. Nine rabbits died within 7 days of vaccination. These rabbits were vaccinated with Filavac K C + V and the datasheet says that the onset of immunity is 7 days post‐inoculation. Another rabbit was vaccinated 10 days before death so it is possible that it had insufficient immunity to overcome infection. The vaccination status of a further rabbit was questionable because there was only a verbal report from the previous owner that the rabbit was vaccinated against RHDV2.

We conclude that RHDV2 is a common cause of sudden death in the UK pet rabbit population, especially in unvaccinated rabbits or those that are only vaccinated against RHDV1. PCR testing complements histopathology in the diagnosis of RHDV2 although false‐negative PCR test results are possible. False‐positives are unlikely. Failure of vaccination against RHDV2 appears rare in rabbits that have received inactivated RHDV2 vaccines, especially if more than 10 days has elapsed since inoculation. Vaccination against both RHDV1 and RHDV2 is still recommended for all pet rabbits.
